# Ammonium tris­(3-amino­pyrazine-2-carboxyl­ato-κ^2^ *N* ^1^,*O*)nickelate(II) trihydrate

**DOI:** 10.1107/S1600536809048363

**Published:** 2009-11-21

**Authors:** Xiao-Li Cheng, Shan Gao, Seik Weng Ng

**Affiliations:** a College of Chemistry and Materials Science, Heilongjiang University, Harbin 150080, People’s Republic of China; b Department of Chemistry, University of Malaya, 50603 Kuala Lumpur, Malaysia

## Abstract

The Ni^II^ atom in the title hydrated salt, (NH_4_)[Ni(C_5_H_4_N_3_O_2_)_3_]·3H_2_O, is *N*,*O*-chelated by the three 3-aminopyrazine-2-carboxyl­ate ligands, resulting in a distorted octa­hedral *mer*-NiN_3_O_3_ geometry for the metal. In the crystal, the complex anion, ammonium cation and uncoordinated water mol­ecules are linked by extensive N—H⋯N, N—H⋯O, O—H⋯N and O—H⋯O hydrogen bonds, forming a three-dimensional network.

## Related literature

For the crystal structure of diaqua­bis(3-amino­pyrazine-2-carboxyl­ato)nickel(II), see: Ptasiewicz-Bak & Leciejewicz (1999 ▶).
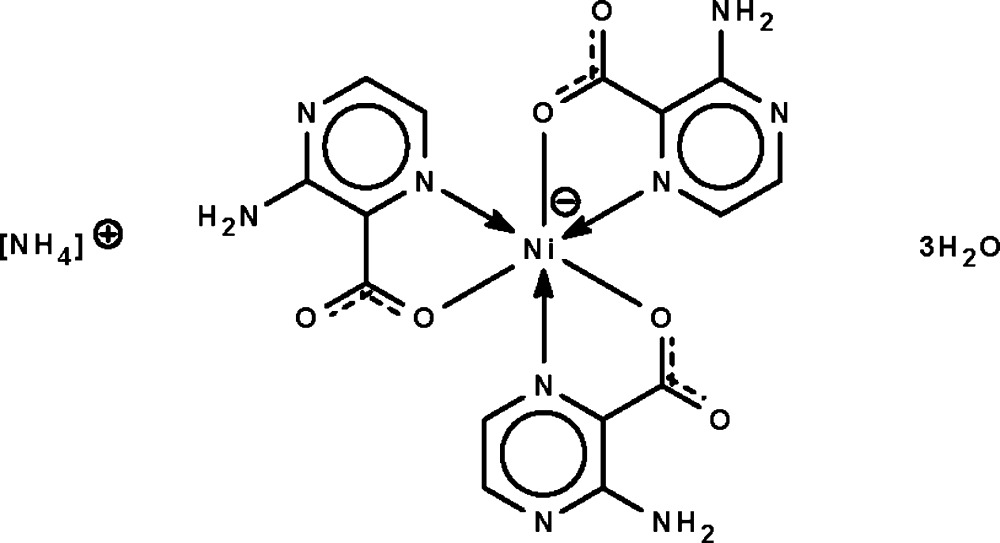


## Experimental

### 

#### Crystal data

(NH_4_)[Ni(C_5_H_4_N_3_O_2_)_3_]·3H_2_O
*M* *_r_* = 545.14
Monoclinic, 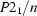

*a* = 11.2092 (3) Å
*b* = 14.7061 (4) Å
*c* = 13.7540 (4) Å
β = 97.5214 (8)°
*V* = 2247.75 (11) Å^3^
*Z* = 4
Mo *K*α radiation
μ = 0.93 mm^−1^
*T* = 293 K
0.28 × 0.22 × 0.19 mm

#### Data collection

Rigaku R-AXIS RAPID IP diffractometer
Absorption correction: multi-scan (*ABSCOR*; Higashi, 1995 ▶) *T*_min_ = 0.780, *T*_max_ = 0.843
21341 measured reflections
5114 independent reflections
4322 reflections with *I* > 2σ(*I*)
*R*_int_ = 0.028

#### Refinement

*R*[*F* ^2^ > 2σ(*F* ^2^)] = 0.035
*wR*(*F* ^2^) = 0.096
*S* = 1.02
5114 reflections
380 parameters
25 restraints
H atoms treated by a mixture of independent and constrained refinement
Δρ_max_ = 0.47 e Å^−3^
Δρ_min_ = −0.26 e Å^−3^

### 

Data collection: *RAPID-AUTO* (Rigaku, 1998 ▶); cell refinement: *RAPID-AUTO* data reduction: *CrystalClear* (Rigaku/MSC, 2002 ▶); program(s) used to solve structure: *SHELXS97* (Sheldrick, 2008 ▶); program(s) used to refine structure: *SHELXL97* (Sheldrick, 2008 ▶); molecular graphics: *X-SEED* (Barbour, 2001 ▶); software used to prepare material for publication: *publCIF* (Westrip, 2009 ▶).

## Supplementary Material

Crystal structure: contains datablocks global, I. DOI: 10.1107/S1600536809048363/hb5228sup1.cif

Structure factors: contains datablocks I. DOI: 10.1107/S1600536809048363/hb5228Isup2.hkl

Additional supplementary materials:  crystallographic information; 3D view; checkCIF report

## Figures and Tables

**Table 1:** Selected bond lengths (Å)

Ni1—O1	2.0476 (13)
Ni1—O3	2.0526 (13)
Ni1—O5	2.0567 (13)
Ni1—N7	2.0561 (14)
Ni1—N4	2.0805 (14)
Ni1—N1	2.0857 (15)

**Table 2:** Hydrogen-bond geometry (Å, °)

*D*—H⋯*A*	*D*—H	H⋯*A*	*D*⋯*A*	*D*—H⋯*A*
N3—H31⋯O2	0.85 (1)	2.08 (2)	2.740 (3)	134 (2)
N6—H61⋯O4	0.85 (1)	2.01 (2)	2.701 (3)	138 (2)
N6—H62⋯N5^i^	0.85 (1)	2.15 (1)	2.992 (2)	175 (2)
N9—H91⋯O6	0.86 (1)	2.07 (2)	2.733 (2)	134 (2)
N9—H92⋯O3^ii^	0.85 (1)	2.10 (1)	2.924 (2)	164 (2)
N10—H101⋯O2	0.86 (1)	1.91 (1)	2.756 (3)	169 (3)
N10—H102⋯O1w	0.86 (1)	1.94 (1)	2.779 (3)	164 (3)
N10—H103⋯O2w	0.85 (1)	2.07 (1)	2.919 (3)	172 (3)
N10—H104⋯N8^iii^	0.84 (1)	2.36 (2)	3.018 (3)	135 (2)
O1w—H1w1⋯O1	0.84 (1)	2.25 (2)	2.964 (3)	143 (4)
O1w—H1w2⋯N2^iv^	0.85 (1)	2.00 (1)	2.842 (3)	171 (4)
O2w—H2w1⋯O5^v^	0.85 (1)	2.03 (1)	2.869 (2)	168 (3)
O2w—H2w2⋯O6^vi^	0.84 (1)	1.96 (1)	2.766 (2)	159 (3)
O3w—H3w1⋯O4	0.85 (1)	2.39 (5)	2.812 (3)	111 (4)

Symmetry codes: (i) 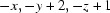; (ii) 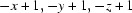; (iii) 
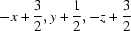
; (iv) 
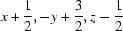
; (v) 
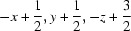
; (vi) 
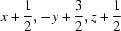
.

**Fig. 1.**
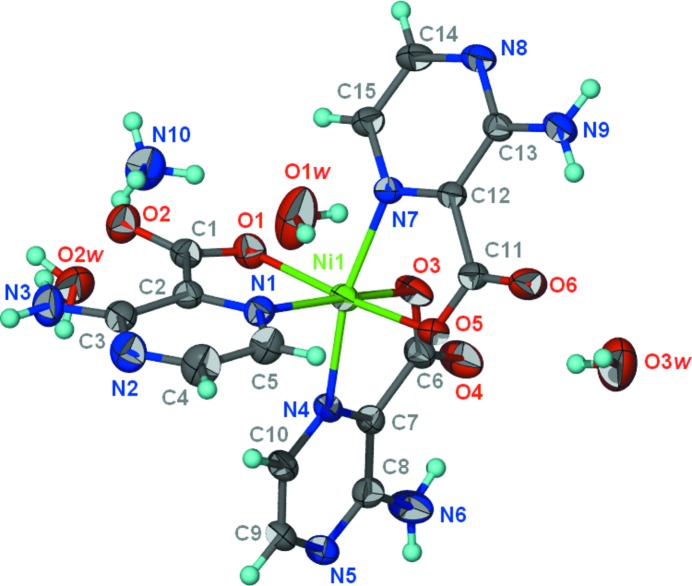
The molecular structure of (I) at the 50% probability level; hydrogen atoms are drawn as spheres of arbitrary radius.

## supplementary crystallographic information

### Experimental

Nickel dichloride hexahydrate (0.48 g, 2 mmol), 3-aminopyrazine-2-carboxylic acid (0.56 g, 4 mmol) and sodium hydroxide (0.16 g 4 mmol) were dissolved in a water/DMF (*v*/*v* = 10 ml:1 ml) mixture. The solution was sealed in a 50 ml Teflon-lined stainless steel bomb and held at 443 K for 3 days. The bomb was gradually cooled to room temperature;, and green blocks of (I) were obtained after several days. The presence of the ammonium counterion is explained by the decomposition of DMF.

### Refinement

Carbon-bound H-atoms were placed in calculated positions (C–H 0.93 Å) and were included in the refinement in the riding model approximation, with *U*(H) = 1.2*U*(C). The amino and water H-atoms were located in a difference Fourier map, and were refined with a distance restraint of N–H = O–H = 0.85±0.01 Å; their U_iso_ values were refined.

### Crystal data

**Table:**

(NH_4_)[Ni(C_5_H_4_N_3_O_2_)_3_]·3H_2_O	*F*(000) = 1128
*M**_r_* = 545.14	*D*_x_ = 1.611 Mg m^−^^3^
Monoclinic, *P*2_1_/*n*	Mo *K*α radiation, λ = 0.71073 Å
Hall symbol: -P 2yn	Cell parameters from 17239 reflections
*a* = 11.2092 (3) Å	θ = 3.2–27.5°
*b* = 14.7061 (4) Å	µ = 0.93 mm^−^^1^
*c* = 13.7540 (4) Å	*T* = 293 K
β = 97.5214 (8)°	Block, green
*V* = 2247.75 (11) Å^3^	0.28 × 0.22 × 0.19 mm
*Z* = 4	

### Data collection

**Table:**

Rigaku R-AXIS RAPID IP diffractometer	5114 independent reflections
Radiation source: fine-focus sealed tube	4322 reflections with *I* > 2σ(*I*)
graphite	*R*_int_ = 0.028
ω scan	θ_max_ = 27.4°, θ_min_ = 3.2°
Absorption correction: multi-scan (*ABSCOR*; Higashi, 1995)	*h* = −14→14
*T*_min_ = 0.780, *T*_max_ = 0.843	*k* = −19→19
21341 measured reflections	*l* = −17→17

### Refinement

**Table:**

Refinement on *F*^2^	Primary atom site location: structure-invariant direct methods
Least-squares matrix: full	Secondary atom site location: difference Fourier map
*R*[*F*^2^ > 2σ(*F*^2^)] = 0.035	Hydrogen site location: inferred from neighbouring sites
*wR*(*F*^2^) = 0.096	H atoms treated by a mixture of independent and constrained refinement
*S* = 1.02	*w* = 1/[σ^2^(*F*_o_^2^) + (0.0582*P*)^2^ + 0.5222*P*] where *P* = (*F*_o_^2^ + 2*F*_c_^2^)/3
5114 reflections	(Δ/σ)_max_ = 0.001
380 parameters	Δρ_max_ = 0.47 e Å^−^^3^
25 restraints	Δρ_min_ = −0.26 e Å^−^^3^

### Fractional atomic coordinates and isotropic or equivalent isotropic displacement parameters (Å^2^)

**Table:**

	*x*	*y*	*z*	*U*_iso_*/*U*_eq_	
Ni1	0.31748 (2)	0.668273 (15)	0.689311 (16)	0.03143 (9)	
O1	0.42705 (12)	0.74401 (9)	0.78914 (10)	0.0399 (3)	
O2	0.46912 (13)	0.77268 (10)	0.94948 (10)	0.0483 (3)	
O3	0.37891 (13)	0.72843 (9)	0.57068 (10)	0.0446 (3)	
O4	0.33775 (17)	0.83556 (12)	0.45711 (12)	0.0616 (5)	
O5	0.21564 (11)	0.57245 (9)	0.60709 (9)	0.0391 (3)	
O6	0.22619 (12)	0.43404 (11)	0.54208 (12)	0.0536 (4)	
O1W	0.5686 (2)	0.8674 (2)	0.67787 (15)	0.0930 (8)	
O2W	0.53958 (16)	1.06663 (15)	0.88906 (13)	0.0643 (5)	
O3W	0.3437 (3)	0.8148 (3)	0.25454 (17)	0.0980 (8)	
N1	0.25466 (14)	0.62854 (10)	0.81904 (11)	0.0341 (3)	
N2	0.15754 (17)	0.62107 (13)	0.99403 (13)	0.0500 (4)	
N3	0.2905 (2)	0.73064 (15)	1.06019 (13)	0.0525 (5)	
N4	0.19233 (13)	0.77229 (10)	0.65903 (10)	0.0327 (3)	
N5	0.03798 (15)	0.91375 (11)	0.59648 (12)	0.0410 (4)	
N6	0.14458 (19)	0.94291 (14)	0.46855 (15)	0.0560 (5)	
N7	0.44529 (13)	0.56953 (10)	0.67977 (10)	0.0307 (3)	
N8	0.60635 (14)	0.44427 (11)	0.62184 (12)	0.0389 (4)	
N9	0.45018 (16)	0.36067 (12)	0.54171 (13)	0.0418 (4)	
N10	0.6367 (2)	0.88322 (17)	0.87878 (16)	0.0609 (5)	
C1	0.40689 (16)	0.73693 (12)	0.87734 (13)	0.0352 (4)	
C2	0.30163 (16)	0.67882 (12)	0.89515 (13)	0.0331 (4)	
C3	0.25094 (18)	0.67630 (13)	0.98471 (14)	0.0389 (4)	
C4	0.1158 (2)	0.56992 (16)	0.91715 (16)	0.0528 (5)	
H4	0.0521	0.5307	0.9229	0.063*	
C5	0.16254 (19)	0.57242 (14)	0.82959 (15)	0.0429 (4)	
H5	0.1305	0.5354	0.7778	0.052*	
C6	0.31612 (18)	0.79401 (13)	0.53101 (13)	0.0385 (4)	
C7	0.21035 (17)	0.82128 (11)	0.58056 (13)	0.0321 (4)	
C8	0.13109 (17)	0.89357 (12)	0.54759 (13)	0.0368 (4)	
C9	0.02365 (18)	0.86309 (14)	0.67384 (14)	0.0413 (4)	
H9	−0.0410	0.8758	0.7077	0.050*	
C10	0.09911 (17)	0.79277 (14)	0.70670 (13)	0.0390 (4)	
H10	0.0855	0.7596	0.7618	0.047*	
C11	0.27182 (16)	0.50055 (13)	0.58777 (13)	0.0345 (4)	
C12	0.40521 (15)	0.49993 (12)	0.62341 (12)	0.0294 (3)	
C13	0.48649 (16)	0.43358 (12)	0.59462 (12)	0.0316 (4)	
C14	0.64164 (17)	0.51541 (14)	0.67792 (15)	0.0418 (4)	
H14	0.7237	0.5235	0.6970	0.050*	
C15	0.56347 (16)	0.57786 (13)	0.70937 (14)	0.0379 (4)	
H15	0.5924	0.6252	0.7507	0.045*	
H1W1	0.518 (4)	0.826 (3)	0.681 (3)	0.16 (2)*	
H1W2	0.587 (4)	0.871 (3)	0.6200 (14)	0.138 (16)*	
H2W1	0.4664 (10)	1.071 (2)	0.8988 (19)	0.079 (10)*	
H2W2	0.586 (2)	1.078 (3)	0.9408 (15)	0.123 (15)*	
H3W1	0.366 (6)	0.861 (2)	0.290 (3)	0.20 (3)*	
H3W2	0.325 (6)	0.772 (3)	0.292 (3)	0.21 (3)*	
H31	0.3539 (16)	0.7617 (16)	1.056 (2)	0.064 (8)*	
H32	0.259 (2)	0.724 (2)	1.1133 (13)	0.076 (9)*	
H61	0.2078 (15)	0.9305 (16)	0.4426 (16)	0.054 (7)*	
H62	0.0924 (18)	0.9821 (13)	0.4466 (18)	0.058 (7)*	
H91	0.3745 (10)	0.3567 (18)	0.5212 (19)	0.059 (7)*	
H92	0.5008 (17)	0.3270 (13)	0.5183 (17)	0.048 (7)*	
H101	0.592 (2)	0.8458 (15)	0.9058 (17)	0.079 (10)*	
H102	0.627 (3)	0.871 (2)	0.8169 (8)	0.106 (13)*	
H103	0.612 (3)	0.9371 (9)	0.887 (2)	0.16 (2)*	
H104	0.7095 (10)	0.876 (2)	0.902 (2)	0.124 (15)*	

### Atomic displacement parameters (Å^2^)

**Table:**

	*U*^11^	*U*^22^	*U*^33^	*U*^12^	*U*^13^	*U*^23^
Ni1	0.03054 (14)	0.03344 (14)	0.03110 (13)	0.00615 (9)	0.00699 (9)	0.00153 (9)
O1	0.0392 (7)	0.0421 (7)	0.0402 (7)	−0.0052 (6)	0.0123 (6)	−0.0010 (6)
O2	0.0463 (8)	0.0536 (8)	0.0440 (7)	−0.0107 (7)	0.0029 (6)	−0.0063 (7)
O3	0.0452 (8)	0.0472 (7)	0.0454 (7)	0.0188 (6)	0.0214 (6)	0.0114 (6)
O4	0.0649 (11)	0.0760 (11)	0.0496 (9)	0.0243 (9)	0.0285 (8)	0.0262 (8)
O5	0.0292 (6)	0.0472 (7)	0.0400 (7)	0.0099 (6)	0.0012 (5)	−0.0059 (6)
O6	0.0326 (7)	0.0540 (9)	0.0713 (10)	0.0016 (6)	−0.0046 (7)	−0.0237 (8)
O1W	0.0979 (17)	0.131 (2)	0.0520 (11)	−0.0539 (16)	0.0174 (11)	−0.0013 (12)
O2W	0.0414 (9)	0.0925 (13)	0.0577 (10)	−0.0038 (9)	0.0021 (8)	−0.0103 (10)
O3W	0.0917 (18)	0.144 (2)	0.0590 (12)	−0.0021 (17)	0.0121 (12)	−0.0216 (15)
N1	0.0345 (8)	0.0350 (8)	0.0325 (7)	0.0019 (6)	0.0037 (6)	0.0027 (6)
N2	0.0528 (11)	0.0581 (11)	0.0419 (9)	−0.0058 (9)	0.0163 (8)	0.0059 (8)
N3	0.0585 (12)	0.0643 (12)	0.0360 (9)	−0.0039 (10)	0.0113 (9)	−0.0049 (9)
N4	0.0335 (8)	0.0342 (7)	0.0312 (7)	0.0067 (6)	0.0067 (6)	−0.0003 (6)
N5	0.0408 (9)	0.0391 (8)	0.0427 (8)	0.0131 (7)	0.0042 (7)	−0.0013 (7)
N6	0.0556 (12)	0.0564 (11)	0.0582 (11)	0.0224 (10)	0.0161 (10)	0.0245 (10)
N7	0.0276 (7)	0.0340 (7)	0.0303 (7)	0.0042 (6)	0.0032 (6)	0.0011 (6)
N8	0.0298 (8)	0.0432 (8)	0.0443 (8)	0.0071 (7)	0.0074 (7)	0.0015 (7)
N9	0.0349 (9)	0.0419 (9)	0.0498 (9)	0.0042 (7)	0.0107 (8)	−0.0092 (8)
N10	0.0507 (12)	0.0779 (16)	0.0551 (12)	−0.0199 (11)	0.0108 (10)	−0.0007 (11)
C1	0.0343 (9)	0.0340 (9)	0.0374 (9)	0.0032 (7)	0.0053 (7)	−0.0006 (8)
C2	0.0325 (9)	0.0349 (9)	0.0317 (8)	0.0058 (7)	0.0037 (7)	0.0029 (7)
C3	0.0408 (10)	0.0432 (10)	0.0329 (9)	0.0067 (8)	0.0054 (8)	0.0042 (8)
C4	0.0518 (13)	0.0584 (13)	0.0501 (12)	−0.0145 (11)	0.0138 (10)	0.0067 (11)
C5	0.0442 (11)	0.0436 (10)	0.0407 (10)	−0.0084 (9)	0.0046 (8)	0.0021 (8)
C6	0.0408 (10)	0.0426 (10)	0.0335 (9)	0.0070 (8)	0.0104 (8)	0.0023 (8)
C7	0.0338 (9)	0.0319 (8)	0.0306 (8)	0.0043 (7)	0.0039 (7)	−0.0011 (7)
C8	0.0387 (10)	0.0340 (9)	0.0368 (9)	0.0043 (8)	0.0014 (8)	0.0007 (7)
C9	0.0376 (10)	0.0478 (11)	0.0393 (9)	0.0113 (9)	0.0084 (8)	−0.0069 (9)
C10	0.0387 (10)	0.0461 (10)	0.0339 (9)	0.0089 (8)	0.0111 (8)	−0.0001 (8)
C11	0.0280 (8)	0.0433 (10)	0.0321 (8)	0.0044 (7)	0.0036 (7)	−0.0003 (8)
C12	0.0269 (8)	0.0330 (8)	0.0288 (8)	0.0038 (7)	0.0050 (6)	0.0027 (7)
C13	0.0315 (9)	0.0335 (8)	0.0306 (8)	0.0042 (7)	0.0076 (7)	0.0037 (7)
C14	0.0253 (9)	0.0500 (11)	0.0496 (11)	0.0033 (8)	0.0027 (8)	−0.0003 (9)
C15	0.0304 (9)	0.0412 (10)	0.0409 (9)	0.0008 (8)	0.0006 (7)	−0.0029 (8)

### Geometric parameters (Å, °)

**Table:**

Ni1—O1	2.0476 (13)	N6—C8	1.332 (3)
Ni1—O3	2.0526 (13)	N6—H61	0.853 (10)
Ni1—O5	2.0567 (13)	N6—H62	0.849 (10)
Ni1—N7	2.0561 (14)	N7—C12	1.326 (2)
Ni1—N4	2.0805 (14)	N7—C15	1.340 (2)
Ni1—N1	2.0857 (15)	N8—C14	1.329 (3)
O1—C1	1.267 (2)	N8—C13	1.356 (2)
O2—C1	1.252 (2)	N9—C13	1.329 (2)
O3—C6	1.274 (2)	N9—H91	0.860 (10)
O4—C6	1.237 (2)	N9—H92	0.848 (10)
O5—C11	1.276 (2)	N10—H101	0.861 (9)
O6—C11	1.237 (2)	N10—H102	0.861 (9)
O1W—H1W1	0.84 (1)	N10—H103	0.853 (9)
O1W—H1W2	0.85 (1)	N10—H104	0.844 (9)
O2W—H2W1	0.85 (1)	C1—C2	1.503 (3)
O2W—H2W2	0.84 (1)	C2—C3	1.423 (3)
O3W—H3W1	0.85 (1)	C4—C5	1.375 (3)
O3W—H3W2	0.86 (1)	C4—H4	0.9300
N1—C2	1.332 (2)	C5—H5	0.9300
N1—C5	1.345 (3)	C6—C7	1.498 (3)
N2—C4	1.332 (3)	C7—C8	1.421 (2)
N2—C3	1.344 (3)	C9—C10	1.374 (3)
N3—C3	1.339 (3)	C9—H9	0.9300
N3—H31	0.854 (10)	C10—H10	0.9300
N3—H32	0.858 (10)	C11—C12	1.511 (2)
N4—C7	1.335 (2)	C12—C13	1.426 (2)
N4—C10	1.339 (2)	C14—C15	1.377 (3)
N5—C9	1.326 (3)	C14—H14	0.9300
N5—C8	1.347 (3)	C15—H15	0.9300
			
O1—Ni1—O3	93.69 (6)	H103—N10—H104	111.9 (14)
O1—Ni1—O5	169.25 (5)	O2—C1—O1	125.01 (18)
O3—Ni1—O5	94.48 (6)	O2—C1—C2	118.53 (16)
O1—Ni1—N7	93.74 (6)	O1—C1—C2	116.43 (16)
O3—Ni1—N7	86.83 (5)	N1—C2—C3	120.28 (17)
O5—Ni1—N7	79.76 (5)	N1—C2—C1	115.18 (16)
O1—Ni1—N4	93.66 (6)	C3—C2—C1	124.54 (17)
O3—Ni1—N4	79.14 (5)	N3—C3—N2	118.03 (19)
O5—Ni1—N4	94.73 (6)	N3—C3—C2	121.75 (19)
N7—Ni1—N4	164.51 (6)	N2—C3—C2	120.16 (18)
O1—Ni1—N1	79.56 (6)	N2—C4—C5	123.1 (2)
O3—Ni1—N1	170.67 (6)	N2—C4—H4	118.5
O5—Ni1—N1	93.05 (6)	C5—C4—H4	118.5
N7—Ni1—N1	99.95 (6)	N1—C5—C4	119.79 (19)
N4—Ni1—N1	94.76 (6)	N1—C5—H5	120.1
C1—O1—Ni1	115.52 (12)	C4—C5—H5	120.1
C6—O3—Ni1	116.28 (12)	O4—C6—O3	124.70 (18)
C11—O5—Ni1	115.38 (11)	O4—C6—C7	119.59 (17)
H1W1—O1W—H1W2	111 (2)	O3—C6—C7	115.71 (16)
H2W1—O2W—H2W2	111 (2)	N4—C7—C8	120.49 (17)
H3W1—O3W—H3W2	108 (2)	N4—C7—C6	115.85 (15)
C2—N1—C5	119.07 (16)	C8—C7—C6	123.65 (17)
C2—N1—Ni1	111.95 (12)	N6—C8—N5	117.76 (17)
C5—N1—Ni1	127.91 (13)	N6—C8—C7	122.34 (18)
C4—N2—C3	117.56 (18)	N5—C8—C7	119.90 (17)
C3—N3—H31	117.1 (19)	N5—C9—C10	123.55 (18)
C3—N3—H32	118 (2)	N5—C9—H9	118.2
H31—N3—H32	124 (3)	C10—C9—H9	118.2
C7—N4—C10	119.01 (15)	N4—C10—C9	119.64 (18)
C7—N4—Ni1	112.91 (12)	N4—C10—H10	120.2
C10—N4—Ni1	128.06 (13)	C9—C10—H10	120.2
C9—N5—C8	117.40 (16)	O6—C11—O5	125.35 (17)
C8—N6—H61	114.4 (17)	O6—C11—C12	119.08 (16)
C8—N6—H62	120.9 (18)	O5—C11—C12	115.57 (16)
H61—N6—H62	125 (2)	N7—C12—C13	120.69 (15)
C12—N7—C15	119.61 (15)	N7—C12—C11	115.47 (15)
C12—N7—Ni1	113.37 (11)	C13—C12—C11	123.71 (16)
C15—N7—Ni1	125.84 (12)	N9—C13—N8	117.77 (16)
C14—N8—C13	117.41 (16)	N9—C13—C12	122.88 (16)
C13—N9—H91	116.9 (18)	N8—C13—C12	119.35 (16)
C13—N9—H92	120.5 (17)	N8—C14—C15	123.63 (17)
H91—N9—H92	121 (2)	N8—C14—H14	118.2
H101—N10—H102	107.1 (13)	C15—C14—H14	118.2
H101—N10—H103	108.6 (14)	N7—C15—C14	119.21 (17)
H102—N10—H103	108.8 (13)	N7—C15—H15	120.4
H101—N10—H104	110.0 (13)	C14—C15—H15	120.4
H102—N10—H104	110.3 (14)		
			
O3—Ni1—O1—C1	170.89 (13)	O1—C1—C2—C3	−167.48 (17)
O5—Ni1—O1—C1	−49.7 (3)	C4—N2—C3—N3	177.4 (2)
N7—Ni1—O1—C1	−102.05 (13)	C4—N2—C3—C2	0.1 (3)
N4—Ni1—O1—C1	91.56 (13)	N1—C2—C3—N3	−175.23 (18)
N1—Ni1—O1—C1	−2.61 (13)	C1—C2—C3—N3	3.6 (3)
O1—Ni1—O3—C6	−96.17 (15)	N1—C2—C3—N2	2.0 (3)
O5—Ni1—O3—C6	90.82 (15)	C1—C2—C3—N2	−179.12 (17)
N7—Ni1—O3—C6	170.28 (15)	C3—N2—C4—C5	−1.0 (3)
N4—Ni1—O3—C6	−3.15 (14)	C2—N1—C5—C4	2.1 (3)
O1—Ni1—O5—C11	−53.7 (3)	Ni1—N1—C5—C4	−164.99 (16)
O3—Ni1—O5—C11	85.68 (13)	N2—C4—C5—N1	0.0 (4)
N7—Ni1—O5—C11	−0.27 (13)	Ni1—O3—C6—O4	−177.33 (18)
N4—Ni1—O5—C11	165.13 (13)	Ni1—O3—C6—C7	3.5 (2)
N1—Ni1—O5—C11	−99.84 (13)	C10—N4—C7—C8	−0.6 (3)
O1—Ni1—N1—C2	8.76 (12)	Ni1—N4—C7—C8	178.20 (13)
O5—Ni1—N1—C2	−179.11 (12)	C10—N4—C7—C6	−179.78 (17)
N7—Ni1—N1—C2	100.76 (12)	Ni1—N4—C7—C6	−1.0 (2)
N4—Ni1—N1—C2	−84.10 (13)	O4—C6—C7—N4	179.16 (19)
O1—Ni1—N1—C5	176.63 (17)	O3—C6—C7—N4	−1.6 (3)
O5—Ni1—N1—C5	−11.24 (17)	O4—C6—C7—C8	0.0 (3)
N7—Ni1—N1—C5	−91.37 (17)	O3—C6—C7—C8	179.19 (17)
N4—Ni1—N1—C5	83.77 (17)	C9—N5—C8—N6	178.60 (19)
O1—Ni1—N4—C7	95.17 (13)	C9—N5—C8—C7	−1.0 (3)
O3—Ni1—N4—C7	2.10 (12)	N4—C7—C8—N6	−178.67 (19)
O5—Ni1—N4—C7	−91.56 (13)	C6—C7—C8—N6	0.5 (3)
N7—Ni1—N4—C7	−23.2 (3)	N4—C7—C8—N5	0.9 (3)
N1—Ni1—N4—C7	174.97 (12)	C6—C7—C8—N5	−179.97 (17)
O1—Ni1—N4—C10	−86.20 (16)	C8—N5—C9—C10	0.8 (3)
O3—Ni1—N4—C10	−179.26 (17)	C7—N4—C10—C9	0.4 (3)
O5—Ni1—N4—C10	87.08 (16)	Ni1—N4—C10—C9	−178.17 (14)
N7—Ni1—N4—C10	155.4 (2)	N5—C9—C10—N4	−0.5 (3)
N1—Ni1—N4—C10	−6.39 (17)	Ni1—O5—C11—O6	177.70 (16)
O1—Ni1—N7—C12	175.68 (12)	Ni1—O5—C11—C12	−3.4 (2)
O3—Ni1—N7—C12	−90.82 (12)	C15—N7—C12—C13	0.5 (2)
O5—Ni1—N7—C12	4.31 (12)	Ni1—N7—C12—C13	168.81 (12)
N4—Ni1—N7—C12	−65.9 (3)	C15—N7—C12—C11	−175.57 (16)
N1—Ni1—N7—C12	95.64 (12)	Ni1—N7—C12—C11	−7.23 (18)
O1—Ni1—N7—C15	−16.83 (15)	O6—C11—C12—N7	−173.77 (17)
O3—Ni1—N7—C15	76.67 (15)	O5—C11—C12—N7	7.2 (2)
O5—Ni1—N7—C15	171.80 (16)	O6—C11—C12—C13	10.3 (3)
N4—Ni1—N7—C15	101.6 (2)	O5—C11—C12—C13	−168.66 (16)
N1—Ni1—N7—C15	−96.87 (15)	C14—N8—C13—N9	−177.44 (18)
Ni1—O1—C1—O2	174.82 (15)	C14—N8—C13—C12	2.5 (3)
Ni1—O1—C1—C2	−3.5 (2)	N7—C12—C13—N9	177.08 (17)
C5—N1—C2—C3	−3.1 (3)	C11—C12—C13—N9	−7.2 (3)
Ni1—N1—C2—C3	166.01 (13)	N7—C12—C13—N8	−2.9 (2)
C5—N1—C2—C1	177.96 (16)	C11—C12—C13—N8	172.81 (16)
Ni1—N1—C2—C1	−12.97 (18)	C13—N8—C14—C15	0.1 (3)
O2—C1—C2—N1	−166.99 (16)	C12—N7—C15—C14	2.1 (3)
O1—C1—C2—N1	11.5 (2)	Ni1—N7—C15—C14	−164.65 (14)
O2—C1—C2—C3	14.1 (3)	N8—C14—C15—N7	−2.5 (3)

### Hydrogen-bond geometry (Å, °)

**Table:**

*D*—H···*A*	*D*—H	H···*A*	*D*···*A*	*D*—H···*A*
N3—H31···O2	0.85 (1)	2.08 (2)	2.740 (3)	134 (2)
N6—H61···O4	0.85 (1)	2.01 (2)	2.701 (3)	138 (2)
N6—H62···N5^i^	0.85 (1)	2.15 (1)	2.992 (2)	175 (2)
N9—H91···O6	0.86 (1)	2.07 (2)	2.733 (2)	134 (2)
N9—H92···O3^ii^	0.85 (1)	2.10 (1)	2.924 (2)	164 (2)
N10—H101···O2	0.86 (1)	1.91 (1)	2.756 (3)	169 (3)
N10—H102···O1w	0.86 (1)	1.94 (1)	2.779 (3)	164 (3)
N10—H103···O2w	0.85 (1)	2.07 (1)	2.919 (3)	172 (3)
N10—H104···N8^iii^	0.84 (1)	2.36 (2)	3.018 (3)	135 (2)
O1w—H1w1···O1	0.84 (1)	2.25 (2)	2.964 (3)	143 (4)
O1w—H1w2···N2^iv^	0.85 (1)	2.00 (1)	2.842 (3)	171 (4)
O2w—H2w1···O5^v^	0.85 (1)	2.03 (1)	2.869 (2)	168 (3)
O2w—H2w2···O6^vi^	0.84 (1)	1.96 (1)	2.766 (2)	159 (3)
O3w—H3w1···O4	0.85 (1)	2.39 (5)	2.812 (3)	111 (4)

Symmetry codes: (i) −*x*, −*y*+2, −*z*+1; (ii) −*x*+1, −*y*+1, −*z*+1; (iii) −*x*+3/2, *y*+1/2, −*z*+3/2; (iv) *x*+1/2, −*y*+3/2, *z*−1/2; (v) −*x*+1/2, *y*+1/2, −*z*+3/2; (vi) *x*+1/2, −*y*+3/2, *z*+1/2.
